# Effect of the thymine‐DNA glycosylase rs4135050 variant on Saudi smoker population

**DOI:** 10.1002/mgg3.590

**Published:** 2019-02-18

**Authors:** Mikhlid Almutairi, Abdullah Mohammad Alhadeq, Rafa Almeer, Mohammed Almutairi, Mohammed Alzahrani, Abdelhabib Semlali

**Affiliations:** ^1^ Zoology Department, College of Science King Saud University Riyadh Kingdom of Saudi Arabia; ^2^ Biology Department, College of Science Al Imam Mohammad Ibn Saud Islamic University (IMSIU) Riyadh Saudi Arabia; ^3^ Groupe de Recherche en Écologie Buccale Université Laval Québec Québec Canada; ^4^ Department of Biochemistry, College of Science King Saud University Kingdom of Saudi Arabia Riyadh

**Keywords:** DNA‐repair, polymorphism, smoking, thymine‐DNA glycosylase

## Abstract

**Background:**

Thymine‐DNA glycosylase (TDG) is an essential DNA‐repair enzyme which works in both epigenetic regulation and genome maintenance. It is also responsible for efficient correction of multiple endogenous DNA lesions which occur commonly in mammalian genomes. Research of genetic variants such as SNPs, resulting in disease, is predicted to yield clinical advancements through the identification of sensitive genetic markers and the development of disease prevention and therapy. To that end, the main objective of the present study is to identify the possible interactions between cigarette smoking and the rs4135050 variant of the TDG gene, situated in the intron position, among Saudi individuals.

**Methods:**

TDG rs4135050 (A/T) was investigated by genotyping 239, and 235 blood specimens were obtained from nonsmokers and smokers of cigarette respectively.

**Results:**

T allele frequency was found which showed a significant protective effect on Saudi male smokers (OR = 0.64, *p* = 0.0187) compared to nonsmoking subjects, but not in female smokers. Furthermore, smokers aged less than 29 years, the AT and AT+TT genotypes decreased more than four times the risk of initiation of smoking related‐diseases compare to the ancestral AA homozygous genotype. Paradoxically, the AT (OR = 3.88, *p* = 0.0169) and AT+TT (OR = 2.86, *p* = 0.0420) genotypes were present at a higher frequency in smoking patients aged more than 29 years as compared to nonsmokers at the same ages.

**Conclusion:**

Depending on the gender and age of patients, TDG rs4135050 may provide a novel biomarker for the early diagnosis and prevention of several diseases caused by cigarette smoking.

## 1 INTRODUCTION

Cigarette smoking (CS) has been identified as the primary risk factor for chronic pulmonary disease initiation (Kopa & Pawliczak, 2018). CS may lead to the development of diseases including periodontal disease, oral tumor, lung tumor (Kheradmand, You, Hee Gu, & Corry, 2017; Seifart & Plagens, 2007; Uppal, Mehndiratta, Mohapatra, Grover, & Puri, 2014), breast tumor (Verde et al., 2016), cardiovascular diseases, and asthma (Kovacs et al., 2012). Somatic mutations may be found in nonmalignant tissues caused by CS (Boran et al., 2017). A recent study reported a strong indication that CS is the key factor of genomic instability and heterogeneity, which may lead to the initiation of diverse types of cancer, such as lung, bladder, and colorectal cancers (Kytola et al., 2017). Furthermore, CS may lead to the development of gene mutations which occurs in cell cycle–control p53 gene (TP53 in humans), which is a major cause of cancer development risk among various ethnic populations(Gibbons, Byers, & Kurie, 2014; Kytola et al., 2017; Liu et al., 2014; Wu et al., 2015).

Numerous toxic compounds are present in cigarettes, including reactive oxygen species which may damage DNA, leading to susceptibility to all types of cancers (Kovacs et al., 2012; Pryor, 1997). DNA repair genes have a fundamental role in maintaining genome integrity by repairing the damaged DNA nucleotides (Sjolund et al., 2014) caused by CS through various DNA repair processes, including the nucleotide excision repair mechanism, the base excision repair (BER) mechanism, and the mismatch repair mechanism (Christmann, Tomicic, Roos, & Kaina, 2003; Yu, Chen, Ford, Brackley, & Glickman, 1999). The BER process repairs DNA damage caused by endogenous and environmental agents. The BER pathway is generally activated via DNA glycosylase enzymes which recognize and excise the mismatched and/or damaged nucleotides (Da, Shi, Ning, & Yu, 2018; Sjolund et al., 2014; Sjolund, Senejani, & Sweasy, 2013). Thymine DNA glycosylase (TDG) begins the BER pathway by cleaving the N‐glycosidic bond between the targeted DNA base and the deoxyribose sugar (Da et al., 2018). The TDG gene in humans is situated on chromosome 12q24.1 and contains 10 exons, having a protein length of 410 amino acids (Cortazar, Kunz, Saito, Steinacher, & Schar, 2007). TDG is well known for its ability to catalyze the deletion of uracil and thymine combined with guanine (Sjolund et al., 2014). It is an essential DNA‐repair enzyme which functions in both epigenetic regulation and genome maintenance (Dodd, Yan, Kossmann, Martin, & Ivanov, 2018). It is also responsible for the efficient correction of multiple endogenous DNA lesions which commonly occur in mammalian genomes (Sjolund et al., 2014). DNA repair is a fundamental process in maintaining the genomic stability of the human genome. Abnormal activity of this process may lead to cancer susceptibility (Huang et al., 2015). Genomic instability caused by DNA lesions may contribute to the inefficiency of DNA repair genes (Paz et al., 2017). Therefore, studying genetic variants such as SNPs and their causes of leading to diseases will likely result in clinical advancements, through the identification of sensitive genetic markers and the development of disease prevention and therapy (Saenko & Rogounovitch, 2018). Although SNPs’ role in leading to diseases development, is not thoroughly understood. They have been widely detected in multiple diseases (Bonassi et al., 2005). Shedding light on the role of SNPs in disease pathogenesis in genes located in the BER pathway will hold particular value, as the BER mechanism is sensitive to diverse endogenous and exogenous factors which may be considered a biomarker of DNA damage (Huang et al., 2015). However, no previous studies evaluated the effect of rs4135050 polymorphic variants on molecular activity of TDG, and subsequent functioning of BER system. Genetic polymorphisms in DNA repair genes such as TDG have been cited as a major influence in developing various types of cancer due to repair genes’ contribution to the modification and alteration of gene functions (de Boer, 2002; Xi, Jones, & Mohrenweiser, 2004). One study described the TDG rs4135113 SNP as ordinarily heterozygous with a minor allele frequency of 10%, commonly detected in African and East Asian individuals (Maiti, Morgan, Pozharski, & Drohat, 2008). This SNP may drive tumorigenesis (Sjolund et al., 2014) and is also associated with esophageal squamous cell carcinoma in the Chinese population (Li et al., 2013).

The main objective of the present study is to identify the possible correlation between CS and genetic polymorphism in TDG rs4135050 among Saudi individuals. As a potential biomarker, this has practical applications not only in the diagnosis of diseases associated with the CS but also in the prevention of CS effects on healthy individuals.

## 2 MATERIALS AND METHODS

### 2.1 Ethical compliance

The study was conducted and approved by an ethical committee of Applied Medical Sciences College, King Saud University (KSU), Riyadh, Kingdom of Saudi Arabia (KSA), (ethical approval reference number CAMS 13/3536).

### 2.2 Specimen collection from participants

The participants were 474 Saudi men and women who visited Aleman Public Hospital in Riyadh, in the Kingdom of Saudi Arabia (KSA) from January 2016 to January 2018. Among them, 239 were nonsmokers, and the other 235 were smokers of cigarette whose ethnicity and age matched the nonsmokers’. The smokers and nonsmokers were interviewed via a self‐completed questionnaire about smoking frequency, smoking status, age, gender, and family history. Self‐reported CS history and medical history, including allergy symptoms and disease, were also obtained from the questionnaire. All procedures were performed according to ethical standards. Exclusion criteria included a history of any kind of inflammatory and/or chronic respiratory disease and family history of cancer. Blood specimens were collected from both groups for genotyping of the TDG gene. A detailed description of the study subjects’ general characteristics is given in Table 1.

**Table 1:** Clinical data of the study participants

Patterns	Control (non smokers) *N* (%)	Smokers *N* (%)
Number of participants	239	235
Age (mean ± *SD*)	27.9 ± 8.63	28.5 ± 5.36
Age (years)		
Less than 29 years	169 (0.71)	143 (0.61)
More than 29 years	70 (0.29)	92 (0.39)
Gender		
Men	142 (0.59)	211 (0.90)
Women	97 (0.41)	24 (0.10)
Years of cigarettes smoking		
>5 years	—	93 (0.40)
≤5 years	—	142 (0.60)
Quantity of cigarettes smoking per day		
≥10	—	125 (0.53)
<10	—	110 (0.47)
Cigarettes smoking within the family		
Yes	78 (0.33)	148 (0.63)
No	161 (0.67)	87 (0.37)
Stop of cigarettes smoking for a period and retuned (quit smoking)		
Yes	—	154 (0.66)
No	—	81 (0.34)
Parents consanguineous		
Yes	92 (0.38)	93 (0.40)
No	147 (0.62)	142 (0.60)

### 2.3 Genomic DNA isolation from blood samples

First, 3‐ml blood specimens were taken from the subjects in tubes, containing an anticoagulant substance such as EDTA (EDTA‐coated tubes). Next, genomic DNA was immediately purified from peripheral lymphocytes (200 μl) with the DNA Blood Mini Kit (QIAGEN) according to standard procedures. The purified DNA samples were then preserved at −80°C until molecular analyses were performed. Finally, a spectrophotometer (Nano Drop 8000, Thermo Fisher Scientific) was used to measure the concentration and purity of the isolated DNA. If the A260/A280 ratio of the purified DNA sample was not between 1.7 and 2.0, the isolated DNA was deemed contaminated and excluded from the study.

### 2.4 TDG SNP selection and genotyping

Before the genotyping assay began, 10 ng of genomic DNA blood specimens were prepared. TDG (Gen Bank reference sequence; NC_000012.12, accession number; NC_000012, and region number; 103965,815...103988878) SNP rs4135050 (A/T) was evaluated and selected from the NCBI database (http://www.ncbi.nlm.nih.gov/snp) based on its location, allele frequency, and role in diseases relevance among diverse ethnic groups. Each sample was genotyped in a 10‐μl reaction using TaqMan assay. A 10‐μl reaction comprised the following components: DNA template (2 μl), 40 × TaqMan^®^ Genotyping SNP Assay (0.2 µl) (Applied Biosystems), TaqMan^®^ Genotyping Master Mix (5.3 µl) (Applied Biosystems), and DNase‐free water (2.5 μl). A negative control was performed by DNA substitution with the equivalent DNase‐free water volume. The DNA was amplified in 96‐well plates under the following PCR cycle conditions: an initial denaturation stage at 95°C for 5 min, followed by a PCR stage of 95°C for 30 s (denaturation) repeated for 40 cycles, 60°C for 30 s (annealing), 72°C for 30 s (extension), and a final extension stage at 72°C for 5 min. PCR was accomplished with a Quant Studio™ 7 Flex Real‐time PCR System (Applied Biosystems) with sequence‐detection software for data analyses.

### 2.5 Statistical methods

All statistical methods employed the Statistical Package for the Social Sciences (SPSS) software (version 16.0, SPSS) and Microsoft Excel. Hardy–Weinberg Equilibrium (HWE) of the SNP genotype distributions in the smoking and nonsmoking groups were assessed by the chi‐squared test. Allele and genotype prevalence were contrasted between the groups by using both the chi‐squared test and Fisher's exact test. Multiple logistic regression analyses were used to determine the odd ratios (ORs), and 95% confidence intervals (CIs) were obtained to examine the correlation strength between TDG SNP and CS. Statistical significance was defined as a probability value of *p*  <  0.05.

## 3 RESULTS

### 3.1 General clinical characteristics of the study participants

Table 1 describes the basic features of the smoking and nonsmoking participants. The population comprised a total of 474 Saudi individuals; 235 male and female CS patients, and 239 male and female nonsmoking controls. No significant differences were found between the two categories in age, gender, and smoking characteristics (see Table 1). In fact, the mean ages of the nonsmoking control group and the smoking patients were almost equal (27.9 ± 8.63 and 28.5 ± 5.36, respectively). The smoking class was divided into those who had smoked cigarettes for more than 5 years (40% of the group) and those who had smoked cigarettes for 5 years or less (60%). The smoking group was further separated into two categories, based on the average daily number of cigarettes smoked. The categories were; smokers who smoked more than 10 cigarettes a day (53%) and those who smoked fewer than 10 cigarettes a day (47%, Table 1). CS within the family was reported in 63% of the smoking group and in 33% of the nonsmoking group. Finally, the percentage of smoking patients who had stopped CS for a period and then, started again was 66%, with parent consanguineous of 40% in tobacco users and 38% in nonsmokers (Table 1).

### 3.2 Genetic variation in TDG SNP rs4135050 with CS

The relation between genetic polymorphism rs4135050 (A/T) of the TDG gene and CS among the smoking and nonsmoking groups of the Saudi Arabian population was investigated by using HWE. The TDG SNP is present in the intron region. Table 2 shows the phenotypic and genotypic distributions of TDG SNP rs4135050 among the smokers and the nonsmoking control group. In this ethnic population, a reference allele of the homozygous ancestral allele was identified to detect the potential associated CS risk. No significant correlations were observed between any smoking behavior and the selected TDG SNPs. The genotypic allocation of this SNP was 9% AA, 36% AT, and 55% TT in smoking patients and 8% AA, 31% AT, and 61% TT in the nonsmoking control group. The T allele showed no difference in the wild‐type A allele frequency between the two subject groups. The T allele was distributed at 73% and 77% among the smokers and the controls, respectively, compared to the A allele reference distribution of 27% in the smokers and 23% in the controls (Table 2). The narrower our confidence interval and the more accurate our results are the more powerful our statistical tests.

**Table 2:** Genotype and allele distribution of thymine‐DNA glycosylase rs4135050 in smokers and controls

Alleles	Controls	Smokers	Odd ratio	95% CI	χ^2^	*p* value
Total	225	227				
AA	17 (0.08%)	20 (0.09%)	Ref			
AT	71 (0.31%)	82 (0.36%)	0.98	0.4776–2.0177	0.0025	0.9599
TT	137 (0.61%)	125 (0.55%)	0.78	0.3888–1.5470	0.5224	0.4698
AT+TT	208 (0.92%)	207 (0.91%)	0.85	0.4309–1.6607	0.2368	0.6265
A	105 (0.23%)	122 (0.27%)	Ref			
T	345 (0.77%)	332 (0.73%)	0.83	0.6128–1.1194	1.5051	0.2199

Note

Ref: Reference allele.

*p* < 0.05.

### 3.3 Frequencies of TDG SNP rs4135050 according to smoking duration

As shown in Table 3, the study population was distributed into 93 long‐term smokers (>5 years) and 142 short‐term smokers (≤5 years) to investigate any association between the selected TDG SNP and duration of smoking (Table 3A,B). The investigation of the allele and genotype frequencies for rs4135050 SNP did not present any relationship with CS in long‐ or short‐term smokers when compared to non‐smokers (Table 3A,B). The genotype frequency was distributed into the following categories of nonsmokers: 8% AA, 31% AT, and 61% TT. However, in long‐term and short‐term smokers, genetic frequencies were 8% and 11% AA, 38% and 36% AT, and 54% and 53% TT, respectively. The allelic allocations of this SNP were 23% A and 77% T in those who had never smoked. Conversely, in long‐term smokers, allelic distributions were 27% A and 73% T, while they were 29% A and 71% T in short‐term smokers (Table 3A,B).

**Table 3:** Comparison of genotype and allele distribution of thymine‐DNA glycosylase rs4135050 in smokers with overall controls depending on different clinical characteristics

Alleles	Total	AA	AT	TT	AT+TT	A	T
A) Patients smoking for >5 years							
Controls	225	17 (0.08)	71 (0.31)	137 (0.61)	208 (0.92)	105 (0.23)	345 (0.77%)
>5 years	91	7 (0.08)	35 (0.38)	49 (0.54)	84 (0.92)	49 (0.27)	133 (0.73%)
OR		Ref	1.20	0.87	0.98	Ref	0.83
95% CI			0.4544–3.1544	0.3397–2.2208	0.3924–2.4511		0.5572–1.2246
χ^2^			0.1328	0.0866	0.0017		0.9061
*p* value			0.7156	0.7685	0.9669		0.3411
B) Patients smoking for ≤5 years							
Controls	225	17 (0.08)	71 (0.31)	137 (0.61)	208 (0.92)	105 (0.23)	345 (0.77)
≤5 years	112	12 (0.11)	40 (0.36)	60 (0.53)	100 (0.89)	64 (0.29)	160 (0.71)
OR		Ref	0.80	0.62	0.68	Ref	0.76
95% CI			0.3465–1.8385	0.2791–1.3792	0.3133–1.4806		0.5293–1.0938
χ^2^			0.2812	1.3892	0.9487		2.1842
*p* value			0.5959	0.2385	0.3301		0.1394
C) Patients smoking ≥10 cigarettes/day							
Controls	225	17 (0.08)	71 (0.31)	137 (0.61)	208 (0.92)	105 (0.23)	345 (0.77)
≥10 cigarettes	93	9 (0.10)	31 (0.33)	53 (0.57)	84 (0.90)	49 (0.26)	137 (0.74)
OR		Ref	0.82	0.73	0.76	Ref	0.85
95% CI			0.3315–2.0520	0.3068–1.7406	0.3271–1.7790		0.5746–1.2601
χ^2^			0.1720	0.5048	0.3946		0.6501
*p* value			0.6783	0.4774	0.5299		0.4201
D) Patients smoking <10 cigarettes/day							
Controls	225	17 (0.08)	71 (0.31)	137 (0.61)	208 (0.92)	105 (0.23)	345 (0.77)
<10 cigarettes	106	10 (0.09)	40 (0.38)	56 (0.53)	96 (0.91)	60 (0.28)	152 (0.72)
OR		Ref	0.96	0.69	0.78	Ref	0.77
95% CI			0.4005–2.2905	0.2998–1.6107	0.3464–1.7774		0.5325–1.1164
χ^2^			0.0094	0.7258	0.3394		1.9012
*p* value			0.9227	0.3943	0.5602		0.1679
E) Male smoker patients							
Controls	130	7 (0.05)	38 (0.29)	85 (0.66)	123 (0.95)	52 (0.20)	208 (0.80)
Males	203	20 (0.10)	74 (0.36)	109 (0.54)	183 (0.90)	114 (0.82)	292 (0.72)
OR		Ref	0.68	0.45	0.52	Ref	0.64
95% CI			0.2648–1.7544	0.1813–1.1109	0.2137–1.2688		0.4409–0.9299
χ^2^			0.6364	3.1212	2.1230		5.5283
*p* value			0.4250	0.0773	0.1451		0.0187*
F) Female smoker patients							
Controls	92	8 (0.09)	32 (0.35)	52 (0.56)	84 (0.91)	48 (0.26)	136 (0.74)
Females	25	1 (0.04)	8 (0.32)	16 (0.64)	24 (0.96)	8 (0.17)	40 (0.83)
OR		Ref	2.00	2.46	2.29	Ref	1.76
95% CI			0.2175–18.3883	0.2858–21.1973	0.2722–19.1920		0.7715–4.0364
χ^2^			0.3872	0.7125	0.6104		1.8449
*p* value			0.5338	0.3986	0.4346		0.1744
G) Patients smoking ≥29 years							
Controls	149	5 (0.03)	52 (0.35)	92 (0.62)	144 (0.97)	62 (0.21)	236 (0.79)
≥29 years	124	14 (0.11)	46 (0.37)	64 (0.52)	110 (0.89)	74 (0.30)	174 (0.70)
OR		Ref	0.32	0.25	0.27	Ref	0.62
95% CI			0.1056–0.9449	0.0852–0.7242	0.0954–0.7803		0.4182–0.9125
χ^2^			4.5566	7.3121	6.5801		5.9052
*p* value			0.0328*	0.0068*	0.0103*		0.0151*
H) Patients smoking ˂29 years							
Controls	72	11 (0.15)	17 (0.24)	44 (0.61)	61 (0.85)	39 (0.27)	105 (0.73)
˂ 29 years	101	6 (0.06)	36 (0.36)	59 (0.58)	95 (0.94)	48 (0.24)	154 (0.76)
OR		Ref	3.88	2.46	2.86	Ref	1.19
95% CI			1.2295–12.2596	0.8444–7.1570	1.0037–8.1219		0.7301–1.9451
χ^2^			5.7103	2.8415	4.1358		0.4926
*p* value			0.0169*	0.0919	0.0420*		0.4828

Note

Ref: Reference allele; OR: odd ratio; CI: confidence interval.

* *p* < 0.05 is considered to be statistically significant.

### 3.4 Relationship between TDG SNP rs4135050 and daily CS

As presented in Table 3C,D, the smoking study subjects were divided into heavy smokers (≥10 cigarettes per day, 125 subjects) and moderate smokers (<10 cigarettes per day, 110 subjects) in order to investigate the relationship between TDG polymorphism allelic, genetic differences and the daily rate of cigarette consumption. The SNP analyses of the TDG gene did not display any statistically significant relationship among the heavy or moderate smokers as compared to the nonsmoking group (Table 3C,D). For example, genotypic allocations were 10% and 9% for the AA reference allele, 33% and 38% for heterozygous AT, and 57% and 53% for double mutant TT in heavy and moderate smokers respectively. This is in contrast to 8% AA, 31% AT, and 61% TT in the control group (Table 3C,D). The allelic frequencies were 23% for the reference allele A and 74% for T alleles in the heavy smokers. The allocations were 28% A and 72% T in the moderate smoker class, and 23% A and 77% T alleles in nonsmoking individuals (Table 3C,D).

### 3.5 Association between TDG SNP rs4135050 and gender in smokers

The results supported a correlation between smoker patient gender and polymorphism rs4135050 in the TDG gene. The prevalence of allele and genotype frequencies of the TDG SNP observed in smoker patients and the nonsmoking control according to gender is described in Table 3E,F. Notably, the T allele presents a significant correlation with a protective effect of CS among male smoking patients (OR = 0.64, CI = 0.4409–0.9299, *p* = 0.0187). However, there is no significant association with the T allele among female smoking patients (Table 3F). By contrast, no correlation was observed between the genotypic frequency of the TDG SNP and CS in either gender. The genotypic allocations of the selected SNP were 10% and 4% for the AA reference allele, 36% and 32% for heterozygous AT, and 54% and 64% for double‐mutant TT in male and female smoking subjects, respectively. In the control group, these values were 5% and 9% for AA, 29% and 35% for AT, and 66% and 56% for TT in male and female populations, respectively (Table 3E,F).

### 3.6 TDG SNP rs4135050 correlation with smoking patient age and other clinical characteristics

One of the most important questions investigated in this work was whether the tested TDG SNP had any links with the age of CS patients in phenotype and genotype variations. To determine this, the nonsmoking and smoking patients were categorized by age, with 92 smokers and 70 nonsmokers aged 29 and older and 143 smokers and 169 nonsmokers below age 29 (Table 1). The results show a connection between the TDG SNP rs4135050 and all allelic and genotypic allocations among older CS patients (≥29 years) in contrast with that of nonsmokers. The AT, TT, and AT+TT genotypes and the T allele presented a significant relationship with protection from the effects of CS among older smoking patients (≥29 years) as contrasted with the nonsmoking population (OR = 0.32, CI = 0.1056–0.9449, *p* = 0.0328 for AT; OR = 0.25, CI = 0.0852–0.7242, *p* = 0.0068 for TT; OR = 0.27, CI = 0.0954–0.7803, *p* = 0.0103 for AT+TT; and OR = 0.62, CI = 0.4182–0.9125, *p* = 0.0151 for T allele, Table 3G). Among CS subjects under 29 years, the AT and AT+TT genotypes of the analyzed SNP show a nearly 4‐fold increase of risk of developing diseases linked to CS and a 3‐fold increase of risk over that of the AA homozygous allele, respectively (AT: OR = 3.88, CI = 1.2295–12.2596, *p* = 0.0169; AT+TT: OR = 2.86. CI = 1.0037–8.1219, *p* = 0.0420; Table 3H). The T allele allocation has no relationship with smoking effects in younger smokers (˂29) as compared to the control population (Table 3H).

Finally, a correlation was sought between SNP rs4135050 of the TDG gene and certain clinical characteristics not previously examined; quitting smoking, family smoking history, and parents consanguineous. The resulting analyses show no connection in genotype and allele variations between groups of CS patients and nonsmoking subjects in these characteristics (Table S1).

#### 3.6.1 Comparison of the allele distribution of TDG rs4135050 between KSA and other populations

A comparison was made between the allele variation of TDG rs413505 in the study population (Saudi Arabian) and that in other populations available in the International Hap Map project study groups (http://hapmap.ncbi.nlm.nih.gov/). The results reveal that the allelic variation for TDG rs413505 is clearly different between the Saudi Arabian population and the populations from the Hap Map project. TDG rs413505 presents a similar allele distribution in our study population which is found in two international Hap Map project populations; CEU, and YRI (Table 4). The allele frequency of this SNP is significantly different in the KSA population, used in this study than in the two international populations, HCB and JPT (Table 4).

**Table 4:** Allele frequency comparison of Thymine‐DNA glycosylase rs 413505 between Saudi Arabian and other populations

Population	Samples (*n*)	A	T	χ^2^	*p* value
Saudi Arabia	225	0.233	0.767		
CEU	120	0.25	0.75	0.1195	0.72960
HCB	90	0	1	00	5.2E‐07*
JPT	90	0	1	00	5.2E‐07*
YRI	120	0.258	0.741	0.2649	0.6068

Note

CEU: Utah residents with Northern and Western European ancestry from the CEPH collection; HCB: Han Chinese in Beijing, China; JPT: Japanese in Tokyo, Japan; YRI: Yoruba in Ibadan, Nigeria.

* *p* < 0.05.

### 3.7 Linkage disequilibrium

**Figure 1:**
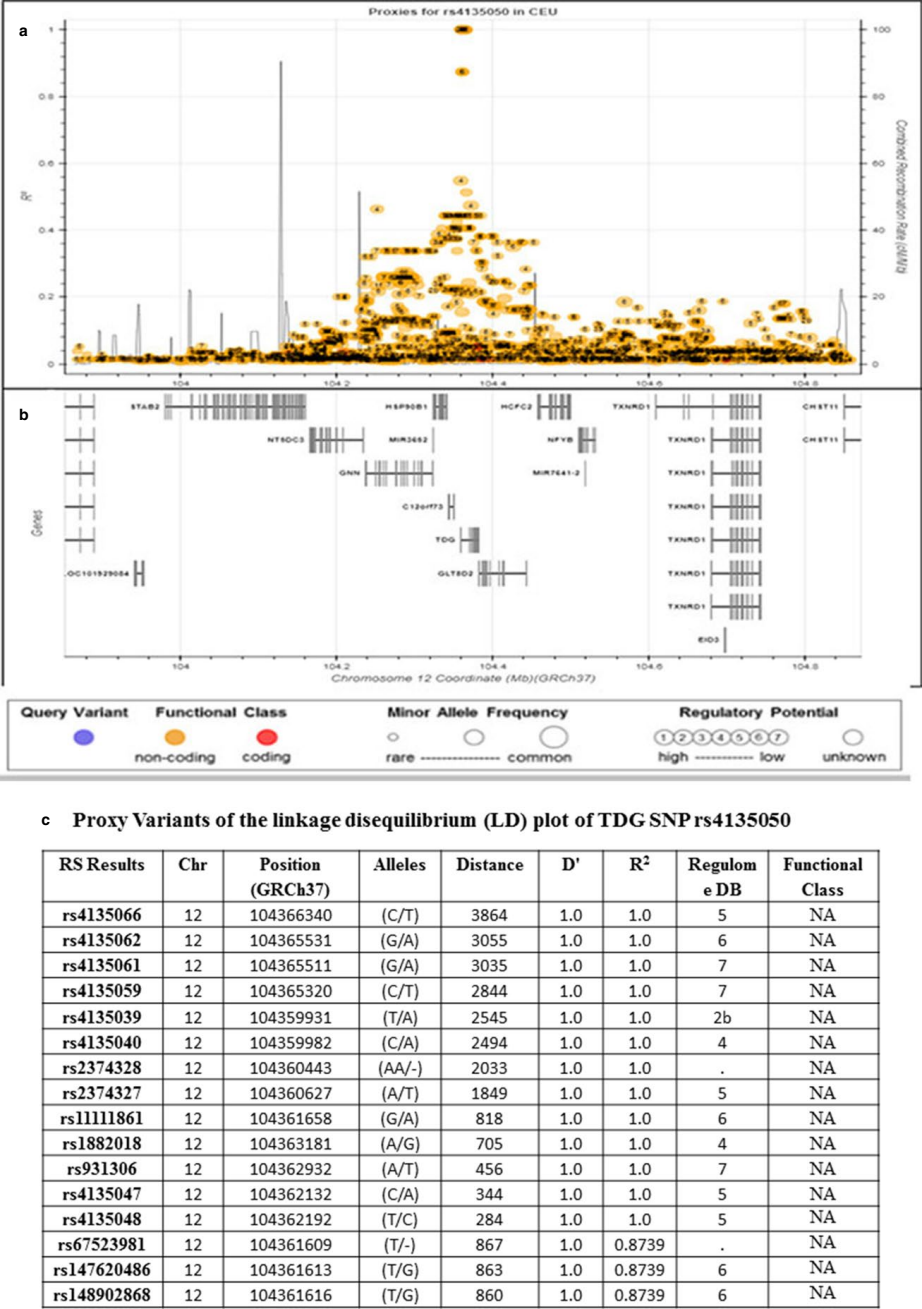
Linkage disequilibrium (LD) plot generated by using interactivity explore Proxy and putatively functional variants (https://ldlink.nci.nih.gov) for the SNP rs4135050. (a) Proxies SNPs for thymine‐DNA glycosylase (TDG) rs 4135050. (b) Proxies genes in chromosome 11 coordinate. (c) Table for proxy variants of LD plot of TDG rs 4135050

One challenge of the current study was to investigate the real association between TDG rs4135050 and a set of SNPs with varying degrees of association due to local linkage disequilibrium (LD) patterns. Genomic regions were visually inspected to determine the extent of their association signal and position, relative to nearby TDG rs4135050 (Figure 1). The analysis reveals that most of the SNP marker combinations exhibited perfect LD scores and show a differential pattern of high LD scores. Figure 1 shows various loci, found very close to the SNP 4135050, with *r* ^2^ values more than 0.8 and up to 1 (Figure1c).

## 4 DISCUSSION

Smoking is a leading cause of 80% of lung cancer, and it also increases the risk of chronic periodontitis (Shereef, Sanara, Karuppanan, Noorudeen, & Joseph, 2015), cancer cell invasion, and metastasis (Liao, Yong, & Hua, 2018). It has also been demonstrated that tobacco smoking causes the development of multiple autoimmune diseases; allergies, chronic pulmonary and vascular, and cancers (Qiu et al., 2017). In recent years, CS has become a major public health issue in the KSA among adolescents (Algorinees et al., 2016), and smokeless tobacco consumption appears to have potential risk factors contributing to oral cancer (Alharbi & Quadri, 2018). The damaging impacts of CS are attributed to the numerous chemical components of cigarettes, such as nicotine and carbon monoxide (Qiu et al., 2017). CS generates DNA damage, leading to mutations and potentially changing the immune microenvironment, which contributes to smoking‐related immune dysfunction (Desrichard et al., 2018). Most DNA mutation, if not repaired, may lead to genetic instability; DNA repair pathways play an essential role in preventing carcinogenesis and maintaining DNA integrity (Kiyohara, Takayama, & Nakanishi, 2006). Genes in DNA repair pathways are vital in protecting DNA from multiple types of damage initiated by tobacco's chemical carcinogens (Hoeijmakers, 2001). Genetic variations, such as SNPs of DNA repair genes, modify DNA repair efficiency by changing protein function and therefore increase the risk for various cancers (de Boer, 2002; Xi et al., 2004), such as chronic pulmonary disease and lung malignancy (Arimilli, Schmidt, Damratoski, & Prasad, 2017; Kheradmand et al., 2017). Among DNA repair genes, the TDG gene was identified as the first mismatch‐specific enzyme playing a key role in recognizing and correcting a variety of damaged and/or mismatched nucleotides (Cortazar et al., 2007; Da & Yu, 2018). All data support the hypothesis that TDG genetic polymorphisms and tobacco smoking may lead to the development of smoking‐related diseases. The main goal of this study was to investigate the potential role of associations between the genetic polymorphism rs4135050 of the TDG gene and CS, using samples from cases and controls in a Saudi Arabian population to detect a genetic marker that could be beneficial to decreasing the risks of disease caused by CS smoking among healthy individuals. A literature review revealed no prior work assessing the relationship between genetic variation of the TDG gene and CS effects. The present study focuses on investigating the allocations of TDG SNP rs4135050 in genomic DNA isolated from the peripheral blood cells of cigarette smokers and nonsmoking subjects. No significant relations between the TDG gene polymorphism tested here and smoking behavior were found in the study population. In addition, no genetic and allelic differences were detected between this SNP and the smoking patients in terms, duration of CS, or daily rate of CS among Saudi smokers as compared to the control subjects. The results suggest that the TDG expression profile is not influenced by the TDG SNP tested here, possibly owing to intron localization of the analyzed polymorphism. Therefore, further studies on the TDG SNPs located in other positions on the gene are strongly recommended, particularly of those SNPs located in the regulatory regions, such as the promoter and exon regions. Specific DNA sequences located in the intron positions, termed the cis‐regulatory elements, may participate in the transcription regulation of gene expression (Jeziorska, Jordan, & Vance, 2009). Similar results were found in previous studies of the TDG, using other polymorphisms in cancer disease among other populations. These polymorphisms were TDG SNP rs4135113, which is unrelated to the risk of skin cancer (Ruczinski et al., 2012), rectal cancer (Curtin et al., 2011), and lung cancer (Krzesniak, Butkiewicz, Samojedny, Chorazy, & Rusin, 2004) and TDG SNP rs2888805, which was reported to have no significant correlation with lung cancer risk (Krzesniak et al., 2004). The present study shows that the smoker's gender plays a major role in the genetic allocations of the TDG gene for rs4135050; in male patients, the T allele for rs413505 presents a significant effect in the prevention of diseases caused by CS. Conversely, in the female population, this SNP appears to increase the protection from CS‐related disease, but not at a statistically significant rate. It should be noted that the number of female samples was insufficient to identify any statistically significant correlation which may exist for this polymorphism in the TDG gene. This limitation was caused by the social traditions in the KSA. Therefore, further study is necessary to confirm these results. The findings of opposite effects of CS by gender are supported by other recent studies. However, it has been found that smoking presents a stronger risk susceptibility of specific cancers in women than in men. Anderson, Moezardalan, Messina, Latreille, and Shaw (2011) has clearly documented that in women, CS can considerably increase the risk for advanced colorectal neoplasia after as little as 10 pack‐years of smoking, whereas it takes 30 or more pack‐years for men (Anderson et al., 2011). This effect is closely related to the effect of CS on sex hormones and seems to vary by menopausal status. Smokers have higher progesterone (Duskova et al., 2012), higher testosterone (Cupisti et al., 2010; Duskova et al., 2012), and lower estrogen levels (Duskova et al., 2012; Gu et al., 2013). The mediating effect of smoking on sex hormones and the subsequent risk of chronic disease, including both cancer and cardiovascular health problems, have attracted growing interest from researchers in recent decades (Benson, Green, Pirie, & Beral, 2010). In a recent study using a sample of nearly 80,000 postmenopausal women, Luo et al. observed an increased risk of breast cancer by 9% and 16% in former smokers and current smokers, respectively, as compared to nonsmokers (Luo et al., 2011). The total number of Saudi smokers among adolescent male increased between 2001 and 2007 (Al‐Bedah, Qureshi, Al‐Guhaimani, & Dukhan, 2011). The present study shows that the age of smokers significantly (*p* < 0.05) affects the relationship between cigarette consumption and the TDG gene for rs4135050. In individuals over 29 years old, the distribution of the AT genotype has protective effects, whereas it has harmful effects in the population under 29 years old. A recent study reveals that the median age of the Saudi population is 30.2 years ("General Authority of Statistics, Kingdome of Saudi Arabia," 2018), and persons of this age—especially males—consume more tobacco products at high amounts since the cost of a pack of 20 cigarettes does not exceed US$2.50. Several epidemiological studies suggest that younger smokers are at greater risk of developing lung cancer and that smoking is more harmful for this age category. Earlier smoking cessation in young adults may bring about greater benefits than in older adults. These results suggest that smoking prevention in young adults should be taken seriously.

## 5 CONCLUSION

The study results show that rs4135050 SNP has a protective effect on older males which could help to inhibit any potential risk of developing smoking‐related disease. In smokers aged less than 29 years, the genotype distribution of this polymorphism presents an increased risk of developing diseases related to CS. Thus, a novel biomarker may exist for the early diagnosis and prevention of several diseases caused by CS in this sub‐category of the population. Further research with sufficiently larger samples, functional analysis, and using various other populations is recommended to verify these findings and to examine the relation between genetic variation of TDG and the effects of smoking.

## CONFLICT OF INTEREST

None declared.

## References

[mgg3590-bib-0001] Al‐Bedah, A. M. , Qureshi, N. A. , Al‐Guhaimani, H. I. , & Dukhan, M. A. (2011). Global health professions student survey in Saudi Arabia. Saudi Medical Journal, 32(6), 636–639. Retrieved from http://www.ncbi.nlm.nih.gov/pubmed/21666949 21666949

[mgg3590-bib-0002] Algorinees, R. M. , Alreshidi, I. G. , Alateeq, M. F. , Alghuraymi, A. A. , Alfayez, A. A. , Almuzaini, F. K. , … Ahmed, H. G. (2016). Prevalence of cigarette smoking usage among adolescent students in Northern Saudi Arabia. Asian Pacific Journal of Cancer Prevention, 17(8), 3839–3843. Retrieved from http://www.ncbi.nlm.nih.gov/pubmed/27644626 27644626

[mgg3590-bib-0003] Alharbi, F. , & Quadri, M. F. A. (2018). Individual and integrated effects of potential risk factors for oral squamous cell carcinoma: A hospital‐based case‐control study in Jazan, Saudi Arabia. Asian Pacific Journal of Cancer Prevention, 19(3), 791–796. 10.22034/APJCP.2018.19.3.791 29582636 PMC5980857

[mgg3590-bib-0004] Anderson, J. C. , Moezardalan, K. , Messina, C. R. , Latreille, M. , & Shaw, R. D. (2011). Smoking and the association of advanced colorectal neoplasia in an asymptomatic average risk population: Analysis of exposure and anatomical location in men and women. Digestive Diseases and Sciences, 56(12), 3616–3623. 10.1007/s10620-011-1814-8 21750931

[mgg3590-bib-0005] Arimilli, S. , Schmidt, E. , Damratoski, B. E. , & Prasad, G. L. (2017). Role of oxidative stress in the suppression of immune responses in peripheral blood mononuclear cells exposed to combustible tobacco product preparation. Inflammation, 40(5), 1622–1630. 10.1007/s10753-017-0602-9 28577134 PMC5587635

[mgg3590-bib-0006] Benson, V. S. , Green, J. , Pirie, K. , & Beral, V. (2010). Cigarette smoking and risk of acoustic neuromas and pituitary tumours in the Million Women Study. British Journal of Cancer, 102(11), 1654–1656. 10.1038/sj.bjc.6605695 20461083 PMC2883161

[mgg3590-bib-0007] Bonassi, S. , Ugolini, D. , Kirsch‐Volders, M. , Stromberg, U. , Vermeulen, R. , & Tucker, J. D. (2005). Human population studies with cytogenetic biomarkers: Review of the literature and future prospectives. Environmental and Molecular Mutagenesis, 45(2–3), 258–270. 10.1002/em.20115 15688363

[mgg3590-bib-0008] Boran, C. , Kandirali, E. , Yanik, S. , Ahsen, H. , Ulukaradag, E. , & Yilmaz, F. (2017). Does smoking change expression patterns of the tumor suppressor and DNA repair genes in the prostate gland? Urologic Oncology: Seminars and Original Investigations, 35(8), 533.e1–533.e8. 10.1016/j.urolonc.2017.03.001 28391998

[mgg3590-bib-0009] Christmann, M. , Tomicic, M. T. , Roos, W. P. , & Kaina, B. (2003). Mechanisms of human DNA repair: An update. Toxicology, 193(1–2), 3–34. 10.1016/S0300-483X(03)00287-7. Retrieved from http://www.ncbi.nlm.nih.gov/pubmed/14599765 14599765

[mgg3590-bib-0010] Cortazar, D. , Kunz, C. , Saito, Y. , Steinacher, R. , & Schar, P. (2007). The enigmatic thymine DNA glycosylase. DNA Repair (Amst), 6(4), 489–504. 10.1016/j.dnarep.2006.10.013 17116428

[mgg3590-bib-0011] Cupisti, S. , Haberle, L. , Dittrich, R. , Oppelt, P. G. , Reissmann, C. , Kronawitter, D. , … Mueller, A. (2010). Smoking is associated with increased free testosterone and fasting insulin levels in women with polycystic ovary syndrome, resulting in aggravated insulin resistance. Fertility and Sterility, 94(2), 673–677. 10.1016/j.fertnstert.2009.03.062 19394003

[mgg3590-bib-0012] Curtin, K. , Ulrich, C. M. , Samowitz, W. S. , Wolff, R. K. , Duggan, D. J. , Makar, K. W. , … Slattery, M. L. (2011). Candidate pathway polymorphisms in one‐carbon metabolism and risk of rectal tumor mutations. International Journal of Molecular Epidemiology and Genetics, 2(1), 1–8. Retrieved from http://www.ncbi.nlm.nih.gov/pubmed/21537397 21537397 PMC3077233

[mgg3590-bib-0013] Da, L. T. , Shi, Y. , Ning, G. , & Yu, J. (2018). Dynamics of the excised base release in thymine DNA glycosylase during DNA repair process. Nucleic Acids Research, 46(2), 568–581. 10.1093/nar/gkx1261 29253232 PMC5778594

[mgg3590-bib-0014] Da, L. T. , & Yu, J. (2018). Base‐flipping dynamics from an intrahelical to an extrahelical state exerted by thymine DNA glycosylase during DNA repair process. Nucleic Acids Research, 46(11), 5410–5425. 10.1093/nar/gky386 29762710 PMC6009601

[mgg3590-bib-0015] de Boer, J. G. (2002). Polymorphisms in DNA repair and environmental interactions. Mutation Research, 509(1–2), 201–210. 10.1016/S0027-5107(02)00217-8. Retrieved from http://www.ncbi.nlm.nih.gov/pubmed/12427539 12427539

[mgg3590-bib-0016] Desrichard, A. , Kuo, F. , Chowell, D. , Lee, K. W. , Riaz, N. , Wong, R. J. , … Morris, L. G. T. (2018). Tobacco smoking‐associated alterations in the immune microenvironment of squamous cell carcinomas. Journal of the National Cancer Institute, 10.1093/jnci/djy060 PMC6292793 29659925

[mgg3590-bib-0017] Dodd, T. , Yan, C. , Kossmann, B. R. , Martin, K. , & Ivanov, I. (2018). Uncovering universal rules governing the selectivity of the archetypal DNA glycosylase TDG. Proceedings of the National Academy of Sciences, 115(23), 5974–5979. 10.1073/pnas.1803323115 PMC6003370 29784784

[mgg3590-bib-0018] Duskova, M. , Simunkova, K. , Hill, M. , Velikova, M. , Kubatova, J. , Kancheva, L. , … Parizek, A. (2012). Chronic cigarette smoking alters circulating sex hormones and neuroactive steroids in premenopausal women. Physiological Research, 61(1), 97–111. Retrieved from http://www.ncbi.nlm.nih.gov/pubmed/22188108 22188108 10.33549/physiolres.932164

[mgg3590-bib-0019] General Authority of Statistics, Kingdome of Saudi Arabia . (2018). Retrieved from https://www.stats.gov.sa/en

[mgg3590-bib-0020] Gibbons, D. L. , Byers, L. A. , & Kurie, J. M. (2014). Smoking, p53 mutation, and lung cancer. Molecular Cancer Research, 12(1), 3–13. 10.1158/1541-7786.MCR-13-0539 24442106 PMC3925633

[mgg3590-bib-0021] Gu, F. Y. , Caporaso, N. E. , Schairer, C. , Fortner, R. T. , Xu, X. , Hankinson, S. E. , … Ziegler, R. G. (2013). Urinary concentrations of estrogens and estrogen metabolites and smoking in caucasian women. Cancer Epidemiology Biomarkers & Prevention, 22(1), 58–68. 10.1158/1055-9965.EPI-12-0909 PMC3643002 23104668

[mgg3590-bib-0022] Hoeijmakers, J. H. (2001). Genome maintenance mechanisms for preventing cancer. Nature, 411(6835), 366–374. 10.1038/35077232 11357144

[mgg3590-bib-0023] Huang, Y. , Li, X. , He, J. , Chen, L. , Huang, H. , Liang, M. , … Xia, T. (2015). Genetic polymorphisms in XRCC1 genes and colorectal cancer susceptibility. World Journal of Surgical Oncology, 13, 244 10.1186/s12957-015-0650-2 26271249 PMC4536607

[mgg3590-bib-0024] Jeziorska, D. M. , Jordan, K. W. , & Vance, K. W. (2009). A systems biology approach to understanding cis‐regulatory module function. Seminars in Cell & Developmental Biology, 20(7), 856–862. 10.1016/j.semcdb.2009.07.007 19660565

[mgg3590-bib-0025] Kheradmand, F. , You, R. , Hee Gu, B. , & Corry, D. B. (2017). Cigarette smoke and DNA cleavage promote lung inflammation and emphysema. Transactions of the American Clinical and Climatological Association, 128, 222–233. Retrieved from http://www.ncbi.nlm.nih.gov/pubmed/28790504 28790504 PMC5525399

[mgg3590-bib-0026] Kiyohara, C. , Takayama, K. , & Nakanishi, Y. (2006). Association of genetic polymorphisms in the base excision repair pathway with lung cancer risk: A meta‐analysis. Lung Cancer, 54(3), 267–283. 10.1016/j.lungcan.2006.08.009 16982113

[mgg3590-bib-0027] Kopa, P. N. , & Pawliczak, R. (2018). Effect of smoking on gene expression profile – overall mechanism, impact on respiratory system function, and reference to electronic cigarettes. Toxicology Mechanisms and Methods, 28(6), 397–409. 10.1080/15376516.2018.1461289 29656668

[mgg3590-bib-0028] Kovacs, K. , Erdelyi, K. , Hegedus, C. , Lakatos, P. , Regdon, Z. , Bai, P. , … Virag, L. (2012). Poly(ADP‐ribosyl)ation is a survival mechanism in cigarette smoke‐induced and hydrogen peroxide‐mediated cell death. Free Radical Biology and Medicine, 53(9), 1680–1688. 10.1016/j.freeradbiomed.2012.08.579 22964577

[mgg3590-bib-0029] Krzesniak, M. , Butkiewicz, D. , Samojedny, A. , Chorazy, M. , & Rusin, M. (2004). Polymorphisms in TDG and MGMT genes – epidemiological and functional study in lung cancer patients from Poland. Annals of Human Genetics, 68(Pt 4), 300–312. 10.1046/j.1529-8817.2004.00079.x 15225156

[mgg3590-bib-0030] Kytola, V. , Topaloglu, U. , Miller, L. D. , Bitting, R. L. , Goodman, M. M. , D`Agostino, R. B. , … Zhang, W. (2017). Mutational landscapes of smoking‐related cancers in Caucasians and African Americans: Precision oncology perspectives at wake forest baptist comprehensive cancer center. Theranostics, 7(11), 2914–2923. 10.7150/thno.20355 28824725 PMC5562225

[mgg3590-bib-0031] Li, W. Q. , Hu, N. , Hyland, P. L. , Gao, Y. , Wang, Z. M. , Yu, K. , … Taylor, P. R. (2013). Genetic variants in DNA repair pathway genes and risk of esophageal squamous cell carcinoma and gastric adenocarcinoma in a Chinese population. Carcinogenesis, 34(7), 1536–1542. 10.1093/carcin/bgt094 23504502 PMC3697889

[mgg3590-bib-0032] Liao, K. , Yong, C. W. , & Hua, K. (2018). SB431542 inhibited cigarette smoke extract induced invasiveness of A549 cells via the TGF‐beta1/Smad2/MMP3 pathway. Oncology Letters, 15(6), 9681–9686. 10.3892/ol.2018.8556 29963124 PMC6020173

[mgg3590-bib-0033] Liu, X. , Lin, X. J. , Wang, C. P. , Yan, K. K. , Zhao, L. Y. , An, W. X. , & Liu, X. D. (2014). Association between smoking and p53 mutation in lung cancer: A meta‐analysis. Clinical Oncology, 26(1), 18–24. 10.1016/j.clon.2013.09.003 24126199

[mgg3590-bib-0034] Luo, J. , Margolis, K. L. , Wactawski‐Wende, J. , Horn, K. , Messina, C. , Stefanick, M. L. , … Rohan, T. E. (2011). Association of active and passive smoking with risk of breast cancer among postmenopausal women: A prospective cohort study. BMJ, 342, d1016 10.1136/bmj.d1016 21363864 PMC3047002

[mgg3590-bib-0035] Maiti, A. , Morgan, M. T. , Pozharski, E. , & Drohat, A. C. (2008). Crystal structure of human thymine DNA glycosylase bound to DNA elucidates sequence‐specific mismatch recognition. Proceedings of the National Academy of Sciences, 105(26), 8890–8895. 10.1073/pnas.0711061105 PMC2449336 18587051

[mgg3590-bib-0036] Paz, M. , de Alencar, M. , Gomes Junior, A. L. , da Conceicao Machado, K. , Islam, M. T. , Ali, E. S. , … de Carvalho Melo‐Cavalcante, A. A. (2017). Correlations between Risk Factors for Breast Cancer and Genetic Instability in Cancer Patients‐A Clinical Perspective Study. Frontiers in Genetics, 8, 236 10.3389/fgene.2017.00236 29503660 PMC5821102

[mgg3590-bib-0037] Pryor, W. A. (1997). Cigarette smoke radicals and the role of free radicals in chemical carcinogenicity. Environmental Health Perspectives, 105(Suppl. 4), 875–882. Retrieved from http://www.ncbi.nlm.nih.gov/pubmed/9255574 9255574 10.1289/ehp.97105s4875 PMC1470037

[mgg3590-bib-0038] Qiu, F. , Fan, P. , Nie, G. D. , Liu, H. , Liang, C. L. , Yu, W. , & Dai, Z. (2017). Effects of cigarette smoking on transplant survival: Extending or shortening it? Frontiers in Immunology, 8, 127 10.3389/fimmu.2017.00127 28239383 PMC5300974

[mgg3590-bib-0039] Ruczinski, I. , Jorgensen, T. J. , Shugart, Y. Y. , Schaad, Y. B. , Kessing, B. , Hoffman‐Bolton, J. , … Alberg, A. J. (2012). A population‐based study of DNA repair gene variants in relation to non‐melanoma skin cancer as a marker of a cancer‐prone phenotype. Carcinogenesis, 33(9), 1692–1698. 10.1093/carcin/bgs170 22581838 PMC3514896

[mgg3590-bib-0040] Saenko, V. A. , & Rogounovitch, T. I. (2018). Genetic polymorphism predisposing to differentiated thyroid cancer: A review of major findings of the genome‐wide association studies. Endocrinology and Metabolism (Seoul), 33(2), 164–174. 10.3803/EnM.2018.33.2.164 PMC6021315 29947173

[mgg3590-bib-0041] Seifart, C. , & Plagens, A. (2007). Genetics of chronic obstructive pulmonary disease. International Journal of Chronic Obstructive Pulmonary Disease, 2(4), 541–550. Retrieved from http://www.ncbi.nlm.nih.gov/pubmed/18268927 18268927 PMC2699975

[mgg3590-bib-0042] Shereef, M. , Sanara, P. P. , Karuppanan, S. , Noorudeen, A. M. , & Joseph, K. (2015). The effect of cigarette smoking on the severity of periodontal diseases among adults of Kothamangalam Town, Kerala. Journal of Pharmacy and Bioallied Sciences, 7(Suppl. 2), S648–651. 10.4103/0975-7406.163588 26538936 PMC4606678

[mgg3590-bib-0043] Sjolund, A. , Nemec, A. A. , Paquet, N. , Prakash, A. , Sung, P. , Doublie, S. , & Sweasy, J. B. (2014). A germline polymorphism of thymine DNA glycosylase induces genomic instability and cellular transformation. PLoS Genetics, 10(11), e1004753 10.1371/journal.pgen.1004753 25375110 PMC4222680

[mgg3590-bib-0044] Sjolund, A. B. , Senejani, A. G. , & Sweasy, J. B. (2013). MBD4 and TDG: Multifaceted DNA glycosylases with ever expanding biological roles. Mutation Research, 743–744, 12–25. 10.1016/j.mrfmmm.2012.11.001 PMC3661743 23195996

[mgg3590-bib-0045] Uppal, V. , Mehndiratta, M. , Mohapatra, D. , Grover, R. K. , & Puri, D. (2014). XRCC‐1 gene polymorphism (Arg399Gln) and susceptibility to development of lung cancer in cohort of north Indian population: A pilot study. Journal of Clinical and Diagnostic Research, 8(11), 17–20. 10.7860/JCDR/2014/10061.5132 PMC4290231 25584213

[mgg3590-bib-0046] Verde, Z. , Santiago, C. , Chicharro, L. M. , Reinoso‐Barbero, L. , Tejerina, A. , Bandres, F. , & Gomez‐Gallego, F. (2016). Effect of genetic polymorphisms and long‐term tobacco exposure on the risk of breast cancer. International Journal of Molecular Sciences, 17(10), 10.3390/ijms17101726 PMC5085757 27754415

[mgg3590-bib-0047] Wu, X. C. , Zheng, Y. F. , Tang, M. , Li, X. F. , Zeng, R. , & Zhang, J. R. (2015). Association between smoking and p53 mutation in oesophageal squamous cell carcinoma: A meta‐analysis. Clinical Oncology, 27(6), 337–344. 10.1016/j.clon.2015.02.007 25736278

[mgg3590-bib-0048] Xi, T. , Jones, I. M. , & Mohrenweiser, H. W. (2004). Many amino acid substitution variants identified in DNA repair genes during human population screenings are predicted to impact protein function. Genomics, 83(6), 970–979. 10.1016/j.ygeno.2003.12.016 15177551

[mgg3590-bib-0049] Yu, Z. , Chen, J. , Ford, B. N. , Brackley, M. E. , & Glickman, B. W. (1999). Human DNA repair systems: An overview. Environmental and Molecular Mutagenesis, 33(1), 3–20. 10.1002/(SICI)1098-2280(1999)33:1lt;3:AID-EM2gt;3.0.CO;2-L. Retrieved from http://www.ncbi.nlm.nih.gov/pubmed/10037319 10037319

