# Supplementary material for: Effect of the thymine‐DNA glycosylase rs4135050 variant on Saudi smoker population
Source: Mol Genet Genomic Med. 2019 Feb 18;7(4):e00590. doi: 10.1002/mgg3.590 (PMC6465727; doi:10.1002/mgg3.590)
Supplement: Supplementary file 1 [file MGG3-7-na-s001.docx]

**Supplementary Table (S1):** Comparison of distribution frequency of TDG rs4135050 genotype in smokers with overall controls depend on the smoking within the family, stop smoking for period and retuned, and parents consanguineous

| **A) Patients with history of smoking within the family** | | | | | | | |
| --- | --- | --- | --- | --- | --- | --- | --- |
| **Alleles** | **total** | **AA** | **AT** | **TT** | **AT+TT** | **A** | **T** |
| Controls | **67** | 5(0.07) | 18(0.27) | 44(0.66) | 62(0.93) | 28(0.21) | 106(0.79) |
| Smokers | **143** | 15(0.11) | 46(0.32) | 82(0.57) | 128(0.89) | 76(0.27) | 210(0.73) |
| OR |  | Ref | 0.85 | 0.62 | 0.69 | Ref | 0.73 |
| 95% CI |  |  | 0.2699-2.6890 | 0.2117-1.8227 | 0.2392-1.9796 |  | 0.4462-1.1941 |
| X^2^ |  |  | 0.0748 | 0.7618 | 0.4851 |  | 1.5790 |
| P value |  |  | 0.7844 | 0.3828 | 0.4861 |  | 0.2089 |
| **B) Patients with no history of smoking within the family** | | | | | | | |
| Controls | **139** | 11(0.08) | 45(0.32) | 83(0.60) | 128(0.92) | 67(0.24) | 211(0.76) |
| Smokers | **84** | 5(0.06) | 36(0.43) | 43(0.51) | 79(0.94) | 46(0.27) | 122(0.73) |
| OR |  | Ref | 1.76 | 1.14 | 1.36 | Ref | 0.84 |
| 95% CI |  |  | 0.5604-5.5273 | 0.3721-3.4913 | 0.4548-4.0535 |  | 0.5443-1.3031 |
| X^2^ |  |  | 0.9532 | 0.0525 | 0.3024 |  | 0.5956 |
| P value |  |  | 0.3289 | 0.8187 | 0.5824 |  | 0.4403 |
| **C) Patients stop smoking for period and retuned** | | | | | | | |
| **Alleles** | **total** | **AA** | **AT** | **TT** | **AT+TT** | **A** | **T** |
| Controls | **225** | 17(0.08) | 71(0.31) | 137(0.61) | 208(0.92) | 105(0.23) | 345(0.77) |
| Smokers | **148** | 16(0.11) | 48(0.32) | 84(0.57) | 132(0.89) | 80(0.27) | 216(0.73) |
| OR |  | Ref | 0.72 | 0.65 | 0.67 | Ref | 0.82 |
| 95% CI |  |  | 0.3310-1.5587 | 0.3125-1.3583 | 0.3293-1.3807 |  | 0.5867-1.1510 |
| X^2^ |  |  | 0.7038 | 1.3201 | 1.1731 |  | 1.3063 |
| P value |  |  | 0.4015 | 0.2506 | 0.2788 |  | 0.2531 |
| **D) Patients do not stop smoking for period and retuned** | | | | | | | |
| Controls | **225** | 17(0.08) | 71(0.31) | 137(0.61) | 208(0.92) | 105(0.23) | 345(0.77) |
| Smokers | **79** | 4(0.05) | 34(0.43) | 41(0.52) | 75(0.95) | 42(0.27) | 116(0.73) |
| OR |  | Ref | 2.04 | 1.27 | 1.53 | Ref | 0.84 |
| 95% CI |  |  | 0.6359-6.5142 | 0.4053-3.9918 | 0.4996-4.7002 |  | 0.5550-1.2731 |
| X^2^ |  |  | 1.4770 | 0.1706 | 0.5648 |  | 0.6733 |
| P value |  |  | 0.2242 | 0.6796 | 0.4523 |  | 0.4119 |
| **E) Patients with parents consanguineous** | | | | | | | |
| **Alleles** | **total** | **AA** | **AT** | **TT** | **AT+TT** | **A** | **T** |
| Controls | **83** | 6(0.07) | 29(0.0.35) | 48(0.58) | 77(0.93) | 41(0.25) | 125(0.75) |
| Smokers | **90** | 9(0.10) | 36(0.40) | 45(0.50) | 81(0.90) | 54(0.30) | 126(0.70) |
| OR |  | Ref | 0.83 | 0.63 | 0.70 | Ref | 0.77 |
| 95% CI |  |  | 0.2639-2.5953 | 0.2059-1.8968 | 0.2384-2.0632 |  | 0.4757-1.2312 |
| X^2^ |  |  | 0.1055 | 0.6968 | 0.4187 |  | 1.2185 |
| P value |  |  | 0.7453 | 0.4039 | 0.5176 |  | 0.2697 |
| **F) Patients without parents consanguineous** | | | | | | | |
| Controls | **137** | 10(0.07) | 40(0.29) | 87(0.64) | 127(0.93) | 60(0.22) | 214(0.78) |
| Smokers | **136** | 11(0.08) | 46(0.34) | 79(0.58) | 125(0.92) | 68(0.25) | 204(0.75) |
| OR |  | Ref | 1.05 | 0.83 | 0.89 | Ref | 0.84 |
| 95% CI |  |  | 0.4021-2.7181 | 0.3326-2.0486 | 0.3670-2.1817 |  | 0.5657-1.2506 |
| X^2^ |  |  | 0.0083 | 0.1714 | 0.0598 |  | 0.7319 |
| P value |  |  | 0.9273 | 0.6789 | 0.8068 |  | 0.3923 |

*P < 0.05 is considered to be statistically significant, Ref = Reference allele
